# Testing the Role of the N-Terminal Tail of D1 in the Maintenance of Photosystem II in Tobacco Chloroplasts

**DOI:** 10.3389/fpls.2016.00844

**Published:** 2016-06-21

**Authors:** Franck Michoux, Niaz Ahmad, Zheng-Yi Wei, Erica Belgio, Alexander V. Ruban, Peter J. Nixon

**Affiliations:** ^1^Sir Ernst Chain Building-Wolfson Laboratories, Department of Life Sciences, Imperial College LondonLondon, UK; ^2^School of Biological and Chemical Sciences, Queen Mary University of LondonLondon, UK

**Keywords:** photosystem II, photoinhibition, PSII repair, chloroplast transformation, d1, D1 degradation

## Abstract

A key step in the repair of photoinactivated oxygen-evolving photosystem II (PSII) complexes is the selective recognition and degradation of the damaged PSII subunit, usually the D1 reaction center subunit. FtsH proteases play a major role in D1 degradation in both cyanobacteria and chloroplasts. In the case of the cyanobacterium *Synechocystis* sp. PCC 6803, analysis of an N-terminal truncation mutant of D1 lacking 20 amino-acid residues has provided evidence that FtsH complexes can remove damaged D1 in a processive reaction initiated at the exposed N-terminal tail. To test the importance of the N-terminal D1 tail in higher plants, we have constructed the equivalent truncation mutant in tobacco using chloroplast transformation techniques. The resulting mutant grew poorly and only accumulated about 25% of wild-type levels of PSII in young leaves which declined as the leaves grew so that there was little PSII activity in mature leaves. Truncating D1 led to the loss of PSII supercomplexes and dimeric complexes in the membrane. Extensive and rapid non-photochemical quenching (NPQ) was still induced in the mutant, supporting the conclusion that PSII complexes are not required for NPQ. Analysis of leaves exposed to high light indicated that PSII repair in the truncation mutant was impaired at the level of synthesis and/or assembly of PSII but that D1 could still be degraded. These data support the idea that tobacco plants possess a number of back-up and compensatory pathways for removal of damaged D1 upon severe light stress.

## Introduction

The multisubunit oxygen-evolving photosystem II (PSII) complex of the thylakoid membrane is susceptible to irreversible damage by light and is considered a weak link in the light reactions of photosynthesis (reviewed by Vass, 2012). PSII activity is maintained through the operation of a PSII repair cycle in which inactivated PSII complexes are partially disassembled and irreversibly damaged PSII subunits, notably the D1 reaction center subunit, are degraded and replaced by newly synthesized subunits; followed by reactivation of PSII activity through light-driven assembly of the inorganic Mn_4_CaO_5_ cluster involved in water oxidation (reviewed by Komenda et al., 2012).

Degradation of photodamaged D1 in the cyanobacterium *Synechocystis* sp. PCC 6803 (hereafter *Synechocystis* 6803) is mediated predominantly by a hexameric FtsH heterocomplex consisting of the FtsH2 and FtsH3 subunits (Silva et al., 2003; Komenda et al., 2006; Boehm et al., 2012). Previous studies on *Escherichia coli* FtsH have concluded that FtsH-catalyzed degradation of membrane proteins is a highly processive reaction usually initiated at the N- or C-terminal tail of a target protein (Chiba et al., 2000, 2002), with efficient degradation at the N-terminus requiring a tail of at least 20 amino-acid residues (Chiba et al., 2000). The observation that shortening the N-terminal tail of D1 to just 12 residues in *Synechocystis* 6803 inhibited D1 degradation during PSII repair provided important evidence that the main pathway for FtsH-mediated proteolysis of damaged D1 proceeded from the N-terminus (Komenda et al., 2007).

Given that FtsH complexes have also been assigned a major role in D1 degradation in chloroplasts (Bailey et al., 2002; Kato et al., 2012), processive N-terminal D1 degradation has likewise been considered a possible mechanism (Nixon et al., 2005; Komenda et al., 2007). Here we have begun to test this hypothesis by using chloroplast transformation technology to generate a tobacco mutant lacking 20 amino-acid residues at the N-terminus of D1. In contrast to the equivalent cyanobacterial mutant, we observe a substantial decrease in PSII accumulation in the mutant. However, D1 could still be degraded in the mutant upon exposure to high light, consistent with the current view that higher plant chloroplasts are able to efficiently remove damaged D1 via multiple pathways depending on the environmental and cellular context.

## Materials and methods

### Growth of plants

Seeds of *Nicotiana tabacum* (cv Petit Havana) were germinated in magenta boxes on Murashige and Skoog (MS) medium containing 8 g L^−1^ agar and 30 g L^−1^ sucrose as described by Ahmad et al. (2012) and plants grown at 25°C, under a day/night cycle of 16 h light/8 h dark, a photon flux density of 50 μmol photons m^−2^ s^−1^ supplied by white fluorescent bulbs and 30% humidity. After 3 or 4 weeks, plants were transferred from MS medium to plastic pots filled with Levington F2 + S seed and modular compost pH 5.3–5.7 (www.scotts.com) supplemented with medium sized Vermiculite pH 6.0 (2–5 mm, density 100 kgm^−3^) (Sinclair, UK) at a ratio of 4:1 and then grown in a greenhouse at 25/20°C (day/night) in a 16 h photoperiod at a photosynthetic photon flux of 120 μmol photons m^−2^ s^−1^ and 40% humidity. The same procedure was adopted for the regeneration of transplastomic mutant plants except that the MS medium contained spectinomycin.

### Generation of transforming plasmids

Total genomic DNA was extracted from tobacco leaves using a DNeasy Plant Mini Kit (PEQLAB, Germany) following the manufacturer's protocol. The transforming plasmid was constructed in four steps using the primers described in Table 1: (1) PCR was performed to amplify a 3-kb genomic fragment between *trnH* and *trnK* (with primers 1 and 2), which was cloned into pGEMT-easy vector (Promega, UK); (2) the *aadA* spectinomycin-resistance cassette was amplified by PCR (using primers 3 and 4) from a modified version of the pHK40 plasmid (Kuroda and Maliga, 2001) in which the tobacco *psbA* promoter from the original pHK40 cassette was replaced by a coffee Prrn16S promoter linked to a T7g10 5′UTR (Michoux, 2008). This modification was performed to avoid any unwanted homologous recombination–mediated rearrangements between the chloroplast transformation vector and the tobacco chloroplast *psbA* or *Prrn* 16S RNA. Construction details are described in Michoux (2008) and are available on request; (3) the amplified *aadA* cassette was inserted into the unique BglII restriction site located at the start of the *psbA* promoter, which had been blunted using Mung Bean nuclease (NEB, UK); (4) the wild-type (WT) BssHII/MfeI fragment encompassing part of the *aadA* and *psbA* genes was replaced by a mutated version lacking 20 codons of *psbA* and now containing a unique NdeI site, generated by overlap extension PCR using primers 5, 6, 7, and 8 (see Table 1 for sequence information). Amplification reactions were performed using Phusion DNA polymerase (Finnzymes, Finland) and the final vector was sequenced to ensure no unwanted DNA mutations have been introduced during the cloning process.

**Table 1:** **Sequence of primers used during PCR reactions**.

**Primer**	**Sequence (5′-3′)**	**Location**
1. B19	AAATCGAATTAAATTCTTCGTTTTTACAAA	27 bp after *trnH*
2. B03	GGGTATCGAACTTCTTAATTGCA	449 bp from end of *matK*
3. aadA-AclI-F	ATCCAACGTTATCGATTTGCTCCCTCAATGAGAATGGAT	5′ start of Prrn16S promoter
4. aadA-AclI-R	TAGAAACGTTACTAGTGGATCGCACTCTACCGATTGA	3′ end of 3′ UTR *rbcL*
5. aadA-BssHII	AGCTAGACAGGCTTATCTTGGACAAGAAGA	116 bp before 3′ end of *aadA* gene
6.PsbA-A20-NdeI-F	AAGATTTTCATATGACTAGCACTGAAAACCGTCTTTACATTGGA	Start codon of *psbA*. New NdeI site shown as underlined
7.PsbA-A20-NdeI-R	TCCAATGTAAAGACGGTTTTCAGTGCTAGTCATATGAAAATCTT	Start codon of *psbA*. New NdeI site shown as underlined
8. PsbA-MfeI	TCCTAGAGGCATACCATCAGAAAAACTTCCT	525 bp after 5′ start of *psbA*

### Generation of tobacco transplastomic mutants

Plastid transformation was performed using the biolistic protocol described by Svab and Maliga (1993). The transformation vector was immobilized onto 550 nm gold particles following the manufacturer's protocol (Seashell Technology, USA). Young leaves of aseptically grown *N. tabacum* were bombarded under sterile conditions on RMOP medium (Svab and Maliga, 1993) using a biolistic device (Bio-Rad Laboratories, UK). Bombarded leaves were then kept in the dark for 48 h at room temperature, before being cut into small pieces and placed on RMOP plates containing spectinomycin (500 mg L^−1^) for regeneration and selection of transformants.

### Evaluation of homoplastomy

After three (03) rounds of selection and regeneration on spectinomycin-containing medium, the putative transformed lines were transferred to compost to evaluate homoplastomy by Southern hybridization using a *psbA*-specific probe and the methods described previously (Ahmad et al., 2012).

### Protein extraction, gel electrophoresis, and immunoblotting

Isolation of thylakoid membrane proteins, quantification of chlorophyll, SDS-PAGE, and Blue-native PAGE (BN-PAGE) were performed as described in Ahmad et al. (2012). Anti-peptide antibodies specific for the C-terminal region of D1 and D2 and antibodies specific for CP43, CP47, and PsaD (Komenda et al., 2007) plus NdhI (Burrows et al., 1998) were used in immunoblotting experiments as described by Ahmad et al. (2012). For relative quantification of D1, signal intensities obtained using ImageJ for 25, 50, and 100% of the untreated sample were used to generate a standard curve (Abramoff et al., 2004). The signal intensities of treated samples were then calculated as a percentage value (of the untreated sample) using this standard curve.

### Fluorescence measurements

For all low temperature fluorescence measurements, leaf homogenates were carefully prepared and diluted to avoid reabsorption (Weis, 1985) in 10 mM Hepes buffer, pH 7.6. Low-temperature (77 K) fluorescence emission spectra were recorded on a Jobin Yvon FluoroMax-3 spectrophotometer equipped with a liquid-nitrogen cooled cryostat. Excitation was defined at 435 nm with a 5-nm spectral bandwidth. The fluorescence spectral resolution was kept at 1 nm. Spectra were normalized at their absolute maximum. The spectral manipulations were performed using GRAMS/AI software (Thermo Fisher Scientific Inc., Waltham, USA).

Chlorophyll fluorescence induction was performed with a DUAL PAM 100 chlorophyll fluorometer (Heinz Walz, Effeltrich, Germany). Plants were adapted in the dark for 30 min before the measurements. Actinic illumination of 100 and 700 μmol photons m^−2^ s^−1^, respectively (see the Results Section), was provided by arrays of 635-nm LEDs illuminating both the adaxial and abaxial surfaces of the leaf. The *F*_o_ (the fluorescence level with open PSII reaction centers) was excited by a measuring beam of 10 μmol photons m^−2^ s^−1^. Maximum fluorescence at the level of all closed reaction centers (*F*_m_) was determined by using a 0.8 s saturating light pulse (10,000 μmol photons m^−2^ s^−1^). The quantum yield of PSII (*F*_v_/*F*_m_) was calculated as [(*F*_m_–*F*_o_)/*F*_m_] and NPQ as [(*F*_m_-$F_{\text{m}}^{\prime }$)/$F_{\text{m}}^{\prime }$], where $F_{\text{m}}^{\prime }$ is the maximum fluorescence level attained at the end of actinic light illumination. *qP* was calculated as [($F_{\text{m}}^{\prime }$-*F*_s_)/*F*_o_], where *F*_s_ is the steady-state fluorescence level at the end of actinic light illumination.

Time-resolved fluorescence spectroscopy was performed using a time-correlated single photon counting (TCSPC) principle on a FluoTime 200 fluorometer (PicoQuant, Berlin, Germany). Detached leaves were vacuum-infiltrated with 50 mM nigericin to completely inhibit NPQ. Excitation at a 10 MHz repetition rate was provided by a 470 nm laser diode, which was carefully adjusted to completely close all PSII reaction centers without causing photoinhibitory quenching of *F*_m_, and to be far below the onset of singlet-singlet exciton annihilation. Fluorescence was detected at 682 nm on leaves with a 2-nm slit width. The instrumental response function was in the range of 50 ps. For lifetime analysis, FluoFit software (PicoQuant, Berlin, Germany) was used. The quality of the fits was judged by the χ^2^ parameter.

### Transmission electron microscopy

Leaf samples obtained from plants growing on sucrose-containing medium were fixed in 2% glutaraldehyde in 0.1 M sodium cacodylate buffer pH 7.2, post-fixed in 1% osmium tetroxide in the same buffer then embedded in epoxy resin. Ultrathin sections were cut and stained with uranyl acetate followed by lead citrate before observation at 120 kV in a FEI Tecnai T12 transmission electron microscope.

### High-light stress experiments

Leaves of mutant and WT plants grown in the greenhouse were placed in darkness overnight, floating in the presence of H_2_O or 5 mM lincomycin in individual petri dishes, with the petiole immersed. The leaves were then exposed to fluorescent light of 1000 μmol photons m^−2^ s^−1^ for up to 4 h, before incubating overnight at 30 μmol photons m^−2^ s^−1^ to test for recovery.

## Results

### Construction of tobacco D1 mutants with truncated N-terminal tail

**Figure 1:**
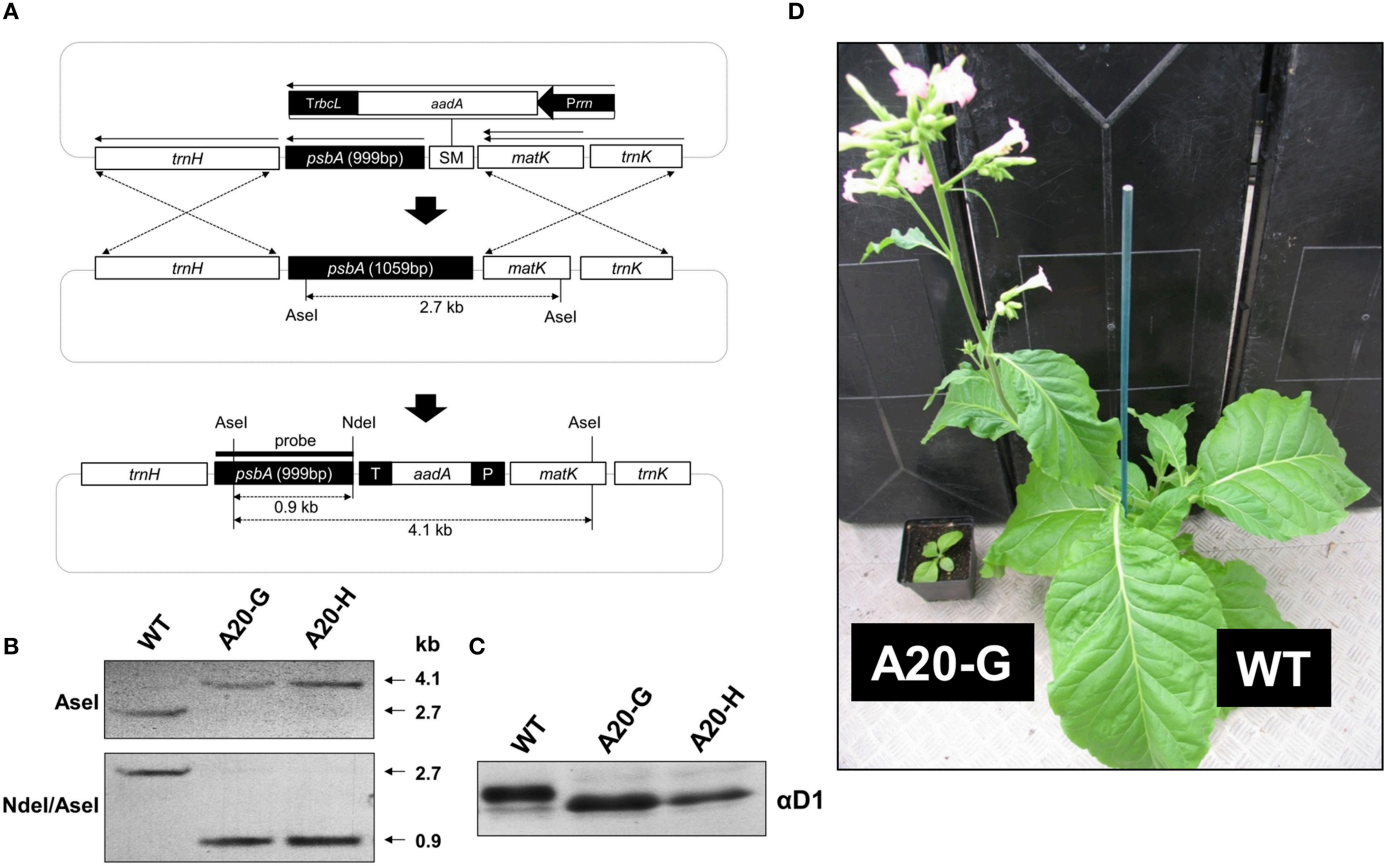
**Construction of tobacco D1 truncation mutants. (A)** Structure of shooting vector showing location of the *aadA* selectable marker (SM) upstream of the *psbA* gene and position of the *psbA* probe. **(B)** Southern hybridization analysis of WT and A20-G and A20-H lines. **(C)** Immunoblot of D1 in WT, A20-G, and A20-H thylakoid membranes carried out on sucrose-grown plants. **(D)** Growth of WT and A20-G in the greenhouse after 4 months.

Previous work has shown that deletion of 20 amino-acid residues from the N-terminal tail of the D1 subunit of the cyanobacterium *Synechocystis* 6803, leaving a predicted tail of 12 residues rather than 32 found in WT, still permitted accumulation of oxygen-evolving complexes. Importantly this *Synechocystis* 6803 mutant (termed A20) was severely impaired in the degradation of D1 during PSII repair. To test whether a similar phenotype would be observed in higher plants, we used chloroplast transformation technology to generate the equivalent mutant in tobacco in which residues D1-T2 to D1-I21, inclusive, were deleted (Figure 1A). Two independent lines, designated A20-G and A20-H, were recovered. Southern blotting indicated that the mutants were homoplastomic (Figure 1B) and immunoblotting experiments confirmed that the D1 protein had a higher electrophoretic mobility in denaturing gels consistent with its smaller size (Figure 1C).

### Tobacco A20 plants accumulate less PSII and grow poorly

**Figure 2:**
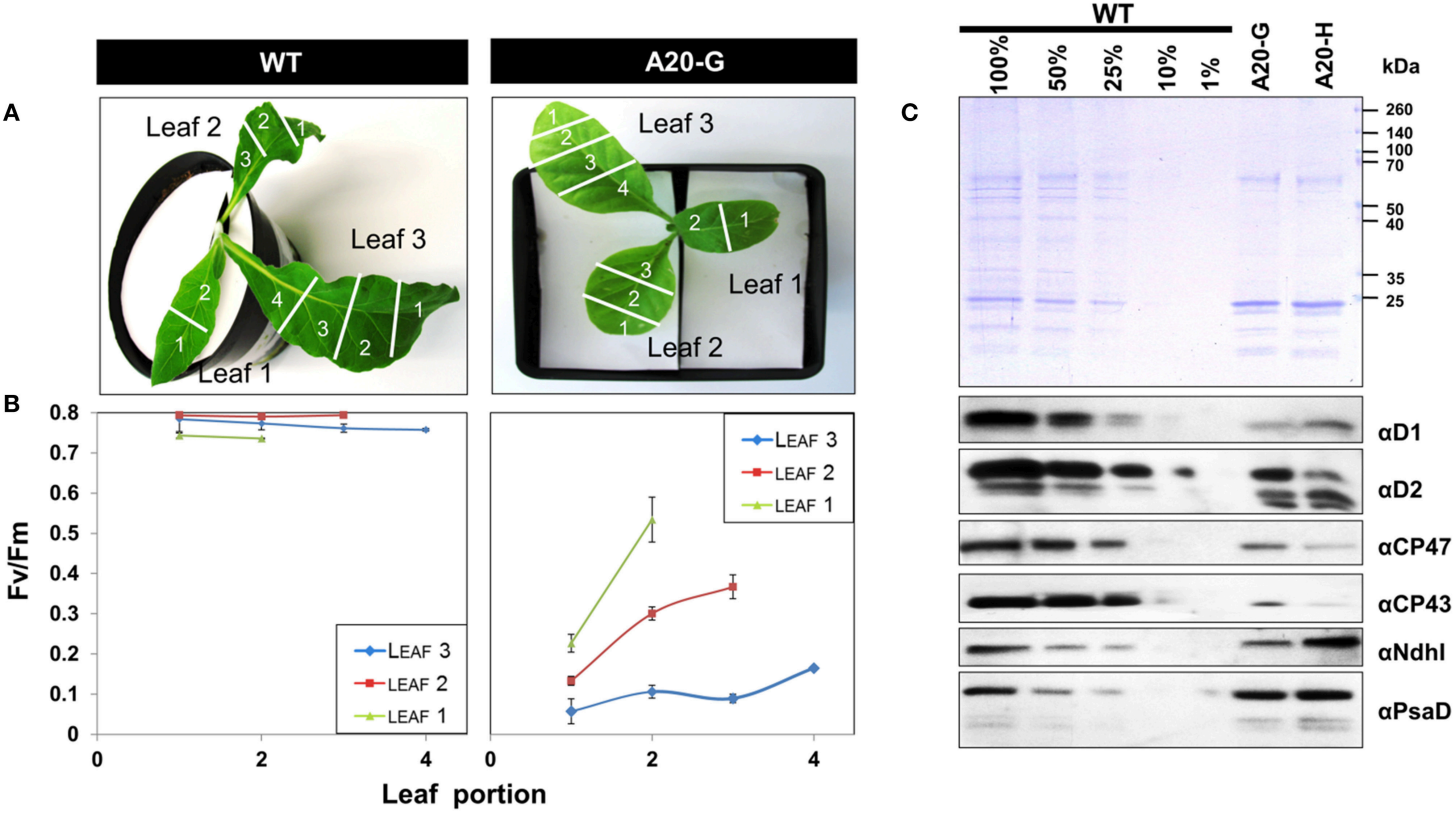
**Reduced photosystem II activity in the mutants. (A,B)** *F*_*v*_/*F*_*m*_ values for various leaf segments of plants grown in compost. **(C)** Immunochemical detection of the D1, D2, CP43, and CP47 subunits of PSII, the NdhI subunit of the NDH complex and the PsaD subunit of PSI. Coomassie-stained gel shows protein loading. Immunoblots were normalized by equal chlorophyll loading and all leaves were pooled for analysis. Plants were ~4–8 weeks old.

**Figure 3:**
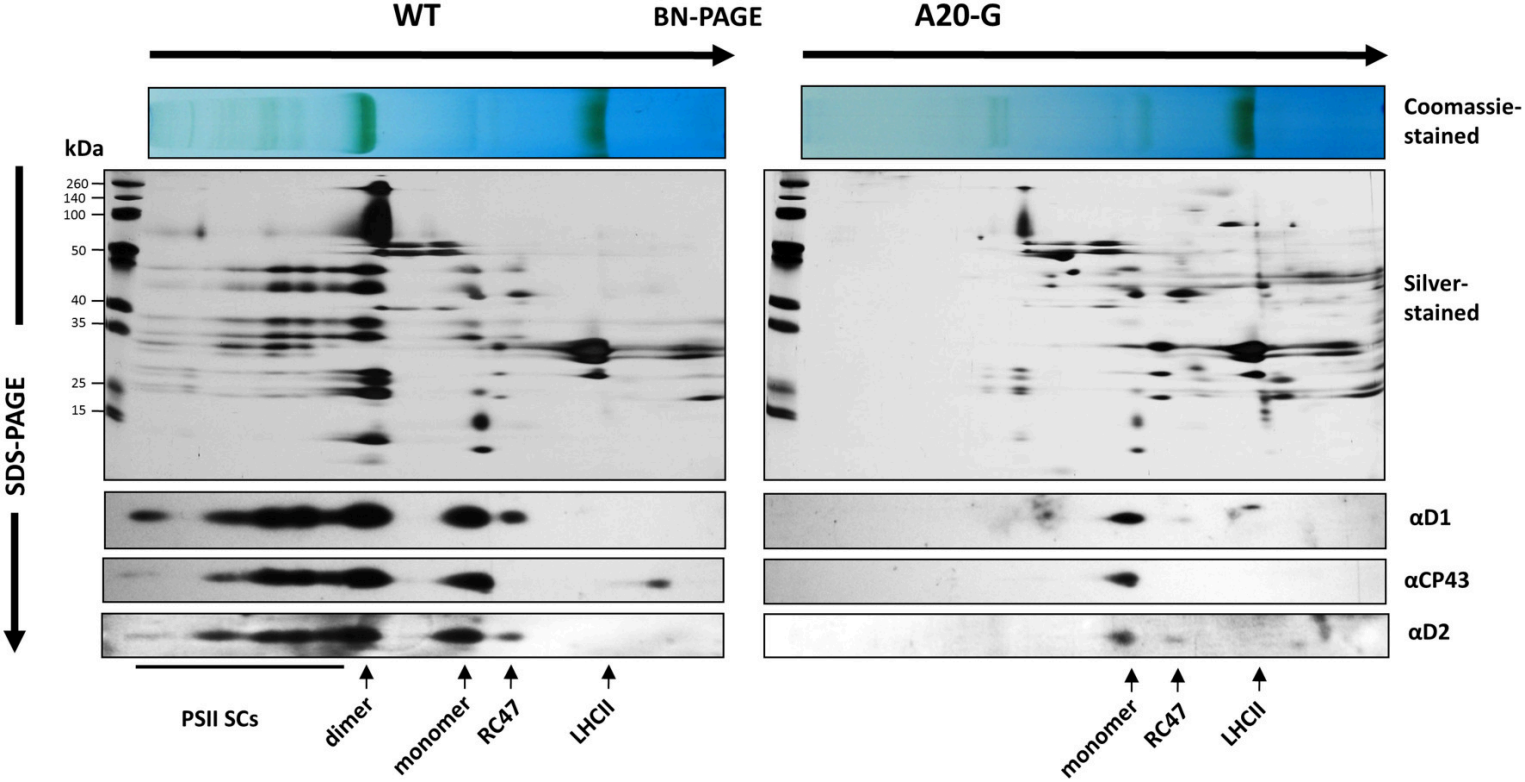
**2D gel analysis of thylakoid proteins**. Detergent-solubilised membrane proteins from either WT or A20-G were separated by BN-PAGE in the first dimension then by denaturing SDS-PAGE in the second dimension followed by immunoblotting using antibodies specific for D1, D2, or CP43 or silver staining. Positions of PSII supercomplexes (PSII SCs), dimeric PSII (dimer), monomeric PSII (monomer), PSII complexes lacking CP43 (RC47), and trimeric LHCII (LHCII) are indicated.

The tobacco A20 mutants showed a clear growth defect when grown photoautotrophically on compost in the greenhouse and were unable to flower and set seed (see Figure 1D for a comparison of A20-G and WT). Determination of the chlorophyll fluorescence parameter (*F*_v_/*F*_m_) in dark-adapted leaves from 6 to 8-week-old plants revealed reduced levels of PSII activity in the A20-G mutant, chosen for further study, with the effect most clearly seen in older leaves (Figures 2A,B). A reduction in the levels of accumulated PSII complex was confirmed by immunoblotting using D1- and D2-specific antibodies (Figure 2C). On an equal chlorophyll basis the mutants only accumulated about 25% of WT levels of PSII whereas levels of photosystem I (PSI) and NADH dehydrogenase-like (NDH) complex were close to that of WT levels as deduced from the PsaD and NdhI immunoblots, respectively. In contrast, control homoplastomic plants containing the spectinomycin-resistance cassette upstream of the intact *psbA* gene contained WT levels of D1 (Supplementary Figure 1) and gave an *F*_*v*_/*F*_*m*_ value indistinguishable from WT (data not shown). Transmission electron microscopy provided evidence that grana could still form in the A20-G mutant but that overall the thylakoid system was less organized than in the WT (Supplementary Figure 2). Analysis of 3-month-old greenhouse-grown material by BN-PAGE revealed that the A20-G mutant only accumulated monomeric PSII complexes and that the PSII-LHCII supercomplexes (PSII SCs) and dimeric PSII complexes observed in WT were undetectable (Figure 3). An intense chlorophyll-containing band corresponding to unassembled LHCII trimers was observed at similar levels in both mutant and WT whereas the mutant extract contained less PSI than WT based on protein staining (Figure 3).

### Tobacco A20 mutant shows enhanced non-photochemical chlorophyll fluorescence quenching

**Figure 4:**
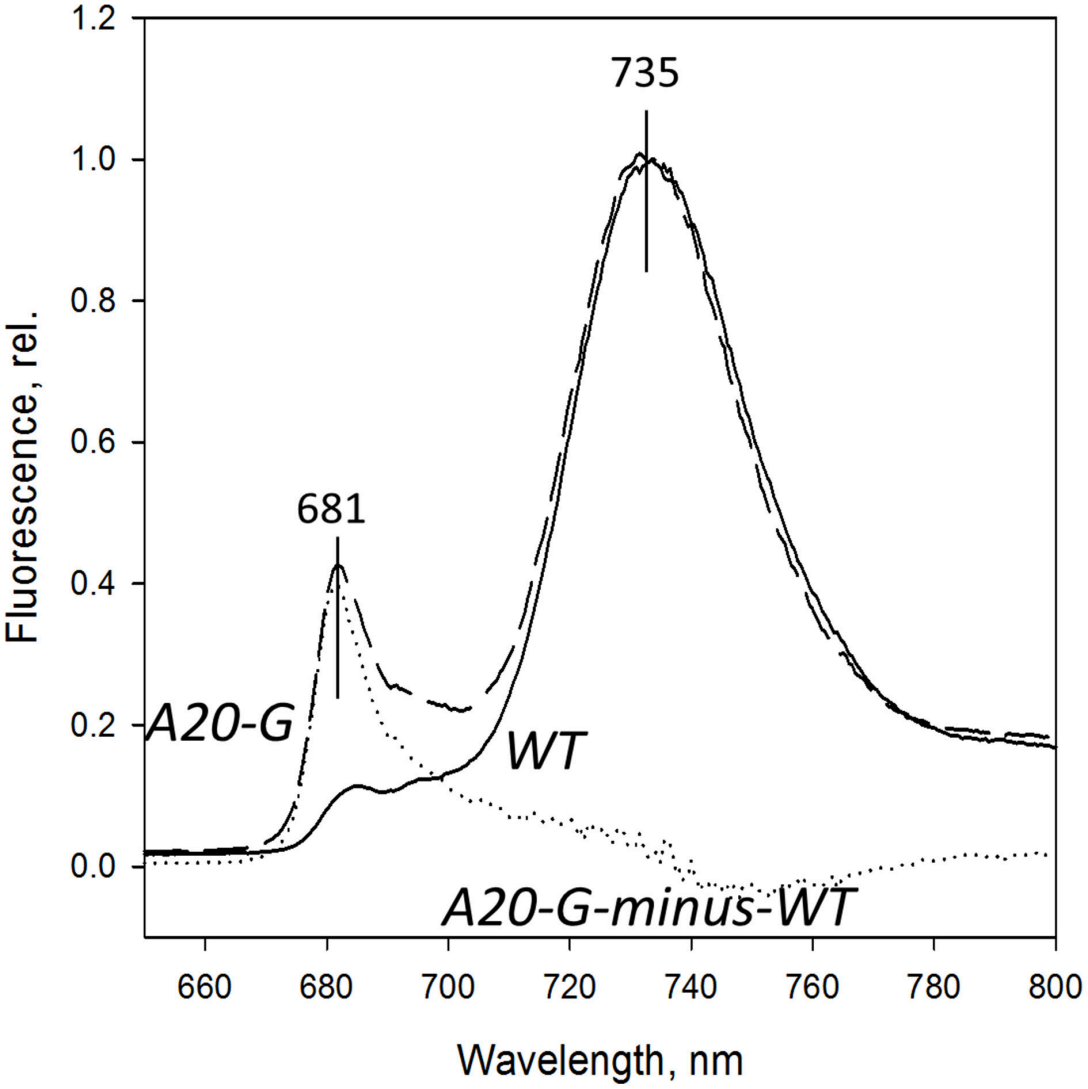
**77K fluorescence emission spectra**. Spectra from chloroplasts of the WT (solid line) and A20-G mutant (dashed line) were normalized at 735 nm. Dashed line is A20-G*minus*-wild type difference spectrum. Chloroplasts were isolated from 3-month-old plants grown in the greenhouse.

Figure 4 shows the 77K fluorescence emission spectra of WT and A20-G leaf homogenates isolated from 3-month-old greenhouse-grown plants normalized to the PSI fluorescence band at 735 nm. The PSII emission band region at 670–700 nm of the mutant revealed strong differences to that of WT. Instead of the two typical bands at 685 and 695 nm originating from the CP43 and CP47 complexes of PSII, respectively (Andrizhiyevskaya et al., 2005), the A20-G mutant possessed one strong band around 681 nm. This emission, which is clearly defined in the A20-G-minus-WT difference spectrum (dotted line), is dominated by the LHCII antenna (Ruban and Horton, 1992). Hence, these data are consistent with the biochemical analysis presented in Figures 2, 3 showing depletion of PSII in the mutant but retention of LHCII.

**Figure 5:**
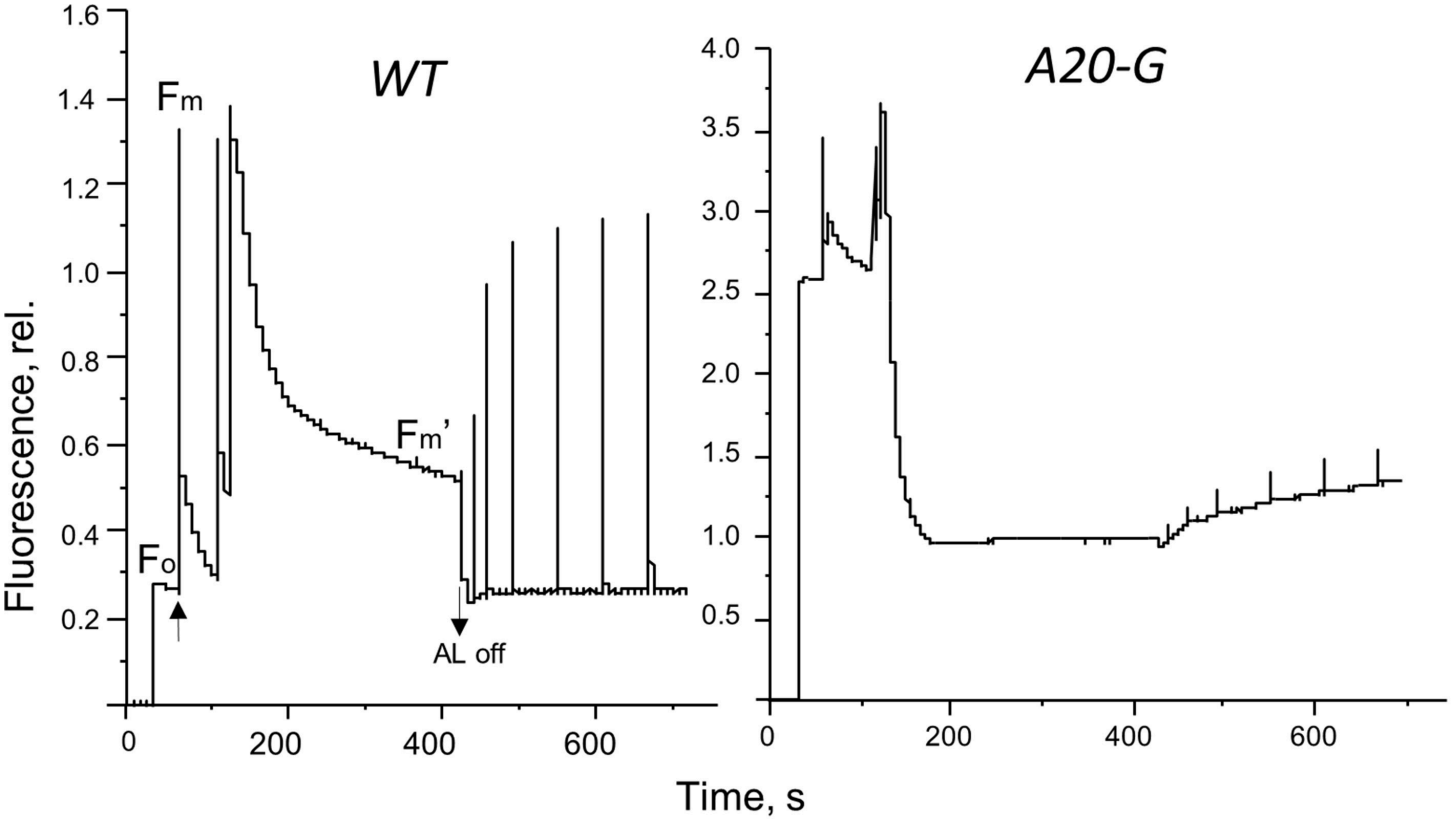
**Fluorescence induction**. Pulse amplitude modulated fluorescence induction traces of leaves from the WT and A20-G mutant of tobacco. Arrows indicate actinic light switched on/off.

**Figure 6:**
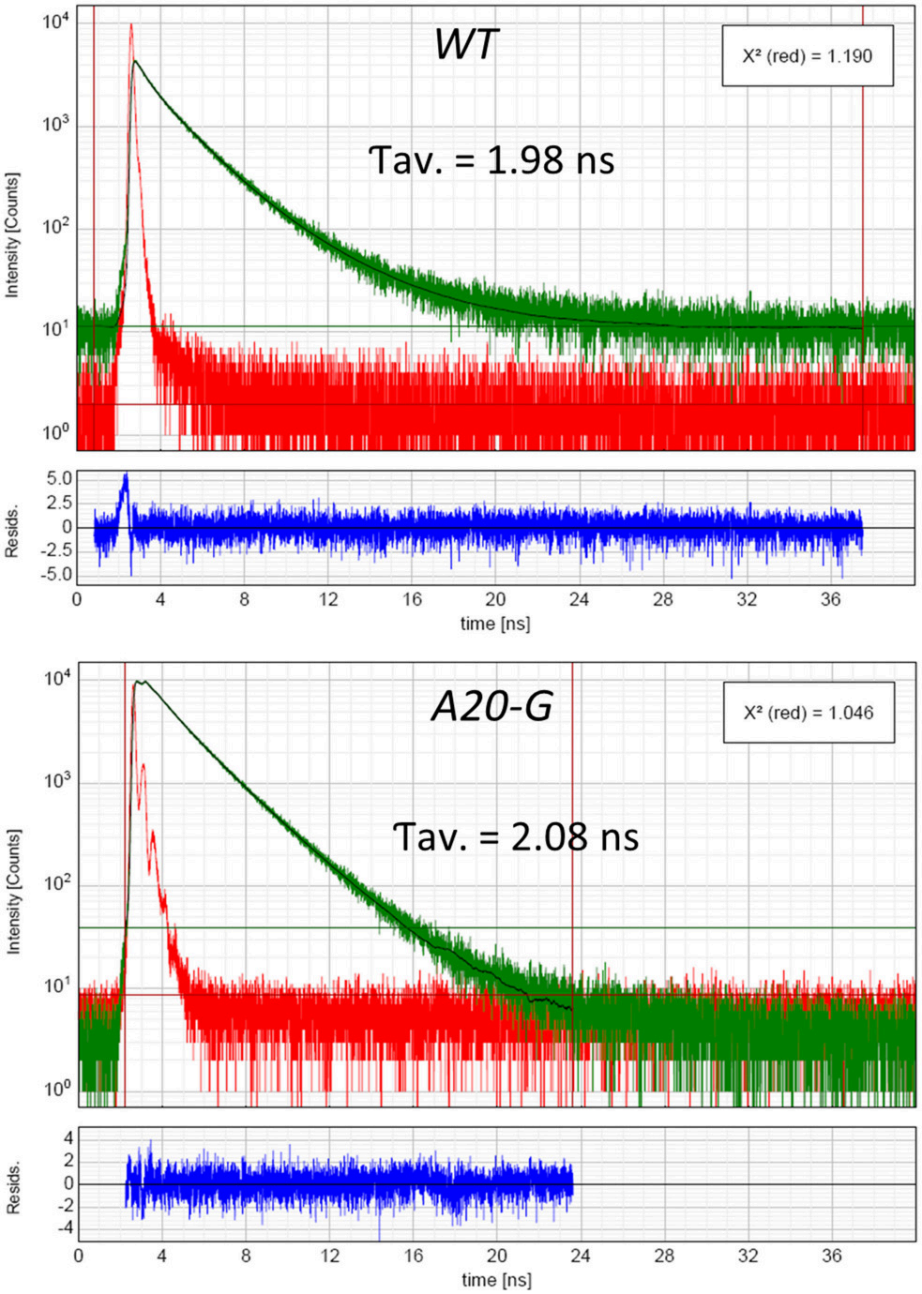
**Lifetime of excited states**. Time-resolved fluorescence analysis of WT and A20-G tobacco leaves measured in *F*_*m*_ state. For more technical details see the Materials and Methods.

The strong increase of LHCII emission indicated that the LHCII complex was not coupled to any reaction center and hence that excitation was not being quenched. Indeed, the PAM fluorescence induction traces in Figure 5 show that the mutant possessed a strongly elevated *F*_*o*_ in comparison to that of the WT and decreased *F*_*v*_/*F*_*m*_ ratio (Table 2). Interestingly, despite vastly reduced levels of PSII in the mutant, illumination induced even more extensive non-photochemical quenching (NPQ) of chlorophyll fluorescence in the mutant than in WT and with much faster kinetics (Figure 5). Relaxation of NPQ in the mutant was, however, slower than in the WT and in the plants illuminated with 700 μmol photons m^−2^ s^−1^ NPQ recovered to only 40% after 30 min in the dark (Table 2). Time-resolved spectroscopy also revealed that the state of LHCII antenna in the A20-G mutant was not affected by the presence of closed reaction centers since the average fluorescence lifetime at *F*_m_ of the mutant plants was almost the same as WT (Figure 6).

**Table 2:** **Fluorescence induction parameters of leaves from WT and A20-G tobacco mutant exposed to two different light intensities**.

**Sample**	**Light Intensity (μmol photons m^−2^ s^−1^)**	***F*_v_/*F*_m_**	***qP***	**NPQ**
WT	100	0.8	0.4	0.96
A20-G	100	0.19	0.07	3.7
WT	700	0.78	0.2	2.1
A20-G	700	0.25	0.003	5.6

F_v_/F_m_ is the PSII fluorescence yield, qP is a photochemical quenching coefficient, and NPQ a measure of non-photochemical quenching.

### D1 is still degraded in the A20 mutant

**Figure 7:**
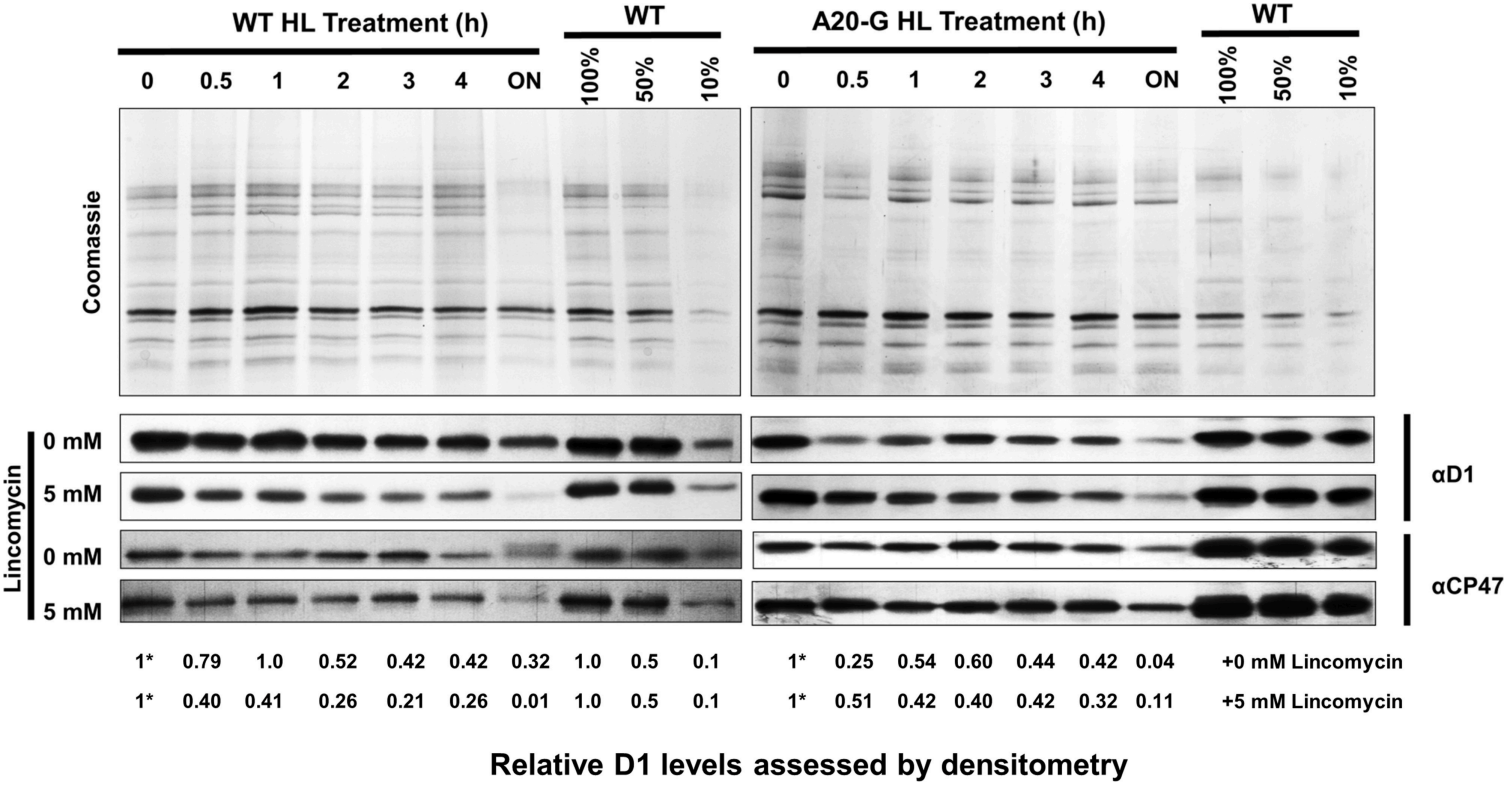
**Analysis of D1 degradation in mutant and WT**. Leaves of WT and A20-G were exposed to high light (HL; 1000 μmol photons m^−2^ s^−1^) for a period of 4 h either in the absence or presence of 5 mM lincomycin then exposed to growth light overnight (ON; 30 μmol photons m^−2^ s^−1^). At each time point, thylakoids were harvested for immunoblotting analysis using D1 and CP47-specific antibodies. Coomassie-stained gels of untreated samples are shown to confirm loading. Relative levels of D1 were determined by densitometry with the initial amount normalized to a value of 1 indicated by asterisk. 3-month-old greenhouse-grown plants were used.

To test the effect of truncating D1 on the degradation of D1, WT, and A20-G leaves were exposed to high light either in the absence or presence of lincomycin, an inhibitor of protein synthesis in the chloroplast, and levels of D1 and CP47 were determined immunochemically. As anticipated from previous work, D1 levels declined in WT leaves in the presence of lincomycin due to light-induced degradation of D1 whereas net loss of D1 was significantly less in the absence of lincomycin due to PSII repair (Figure 7; Kato et al., 2009). In contrast, the low levels of D1 detected in the A20-G leaves declined in both the presence or absence of lincomycin, indicating that degradation of truncated D1 still occurred in the mutant under these illumination conditions; instead the mutant was impaired in the ability to maintain levels of D1 in the absence of lincomycin, most likely due to a defect in the synthesis of D1 and/or its insertion into PSII during the repair process (Figure 7).

## Discussion

We describe here the construction and initial characterization of tobacco mutants designed to test the role of the N-terminal tail of D1 on selective D1 degradation during PSII repair. The mutants were severely impaired in their ability to grow photoautotrophically on compost and there was a loss of PSII activity in leaves as they matured so that mature leaves contained little PSII activity. Loss of PSII subunit expression in older leaves has also been reported in studies on tobacco null mutants lacking low-molecular-mass subunits of PSII and has been ascribed to developmental effects on gene expression (Suorsa et al., 2004). In the case of the tobacco A20 mutants described here, specific effects on the accumulation of *psbA* mRNA, translation efficiency, targeting of D1 to the thylakoid membrane, and assembly into PSII must also be considered. Cyclic electron flow (CEF) around PSI presumably still produced ATP to maintain cellular function in older leaves, although the level of PSI also seemed to decline in the mutant compared to WT as leaves became older (compare Figures 2, 3), possibly in response to reduced levels of PSII activity or differences in chloroplast development.

A dramatic effect seen in the tobacco A20 mutant is the loss of PSII SCs in the membrane as deduced from BN-PAGE (Figure 3) and fluorescence emission spectroscopy (Figure 4), despite the retention of LHCII complexes and some residual PSII. Current structural models of the abundant C_2_M_2_S_2_ PSII SC, which consists of a dimeric PSII core complex surrounded by monomeric (CP29, CP26, and CP29) and trimeric LHCII complexes (Caffarri et al., 2009) place the N-terminal tail of D1 in one PSII monomer in the vicinity of the N-terminal tail of PsbH and CP29 in the opposing monomer of the dimer (Puthiyaveetil and Kirchoff, 2013). Consequently it is possible that truncation of the N-terminal tail might have a direct effect on assembly of dimeric core complexes and larger PSII SCs. However, loss of PSII SCs has been observed in many types of mutant so indirect effects of truncating D1 such as long range effects on the binding of lumenal extrinsic proteins (Ido et al., 2009), low-molecular-mass PSII subunits (Suorsa et al., 2004), or assembly factors such as PsbN (Torabi et al., 2014) are possible. Given the impaired PSII assembly displayed by the mutant, downregulation of PSII activity to minimize damage to PSII or the accumulation of disassembled damaged complexes could also lead to fewer PSII SCs.

A clear phenotype displayed by the tobacco A20 mutants is the ability to perform effective NPQ despite severe depletion of PSII complexes; this phenotype is consistent with energy-dependent qE quenching being a phenomenon of unassembled LHCII complexes as advocated by Ruban and co-workers from analysis of lincomycin-poisoned plant leaves (Belgio et al., 2012).

Our data clearly show that D1 can still be effectively degraded in the A20-G mutant (Figure 7) despite having an N-terminal tail of 12 residues, which is considered too short to engage productively with FtsH complexes in the membrane (Chiba et al., 2000; Lee et al., 2011). Assuming that N-terminal FtsH-mediated D1 degradation is blocked in the A20-G mutant, which is reasonable given the currently accepted mechanism of FtsH, our data support the existence of additional and/or compensatory pathways for D1 degradation. In the literature, discussion has focused on Deg protease-mediated cleavage of D1 coupled to FtsH proteolysis as a supplementary escape pathway that becomes more important at high light intensities (Kato et al., 2012). Kato and colleagues have also provided evidence that depletion of FtsH in the chloroplast leads to the up-regulation of other chloroplast proteases such as Clp and SppA that might compensate for the loss of FtsH-mediated degradation of D1 (Kato et al., 2012). Adam and colleagues have proposed that proper post-translational maturation of the N-terminal tail of D1 is important for recognition by FtsH and that if the N-terminal Met is no longer excised damaged D1 is degraded by an alternative protease(s) (Adam et al., 2011). Additional proteases might also be present at different stages of chloroplast development, such as during senescence.

In the case of *Synechocystis* 6803, D1 degradation in WT appears to occur primarily via the N-terminal FtsH-mediated route (Komenda et al., 2007). However, it must be emphasized that this conclusion is based on the study of D1 degradation under largely non-photoinhibitory conditions when the rates of PSII damage and repair are balanced (Komenda et al., 2007), rather than under the more extreme photoinhibitory conditions usually used to study D1 degradation in plants, that could lead to more extensive photooxidative damage and trigger additional alternative “back-up” pathways to FtsH for D1 degradation. Indeed detailed experiments by Kato et al. (2009) have shown that an Arabidopsis mutant depleted in FtsH is impaired in D1 degradation at low (20 μmol photons m^−2^ s^−1^) to medium irradiances (100 μmol photons m^−2^ s^−1^) but less so at high irradiances (1200 μmol photons m^−2^ s^−1^). Likewise, in the case of *Synechocystis* 6803, it is likely that when FtsH-mediated PSII repair alone cannot cope with the rates of damage to PSII, other proteases might also contribute to D1 degradation as discussed by Nixon et al. (2005). In support of this concept, analysis of cyanobacterial PSII mutants that are more susceptible to photodamage has provided evidence that other proteases can indeed partially compensate for the loss of the FtsH2/FtsH3 complex (Komenda et al., 2010).

## Author contributions

FM, AR, and PN designed the research; FM, NA, ZW, and EB performed the research; All authors analyzed the data FM, NA, AR, and PN wrote the manuscript.

## Funding

We are grateful to the Biotechnology and Biological Science Research Council (grant BB/E006388/1) for supporting this work.

### Conflict of interest statement

The authors declare that the research was conducted in the absence of any commercial or financial relationships that could be construed as a potential conflict of interest.

## References

[B1] Abramoff M. D. Magelhaes P. J. Ram S. J. (2004). Image processing with image. *J*. Biophotonics Int. 11, 36–42.

[B2] Adam Z. Frottin F. Espagne C. Meinnel T. Giglione C. (2011). Interplay between N-terminal methionine excision and FtsH protease is essential for normal chloroplast development and function in Arabidopsis. Plant Cell 23, 3745–3760. 10.1105/tpc.111.087239 22010036 PMC3229147

[B3] Ahmad N. Michoux F. Nixon P. J. (2012). Investigating the production of foreign membrane proteins in tobacco chloroplasts: expression of an algal plastid terminal oxidase. PLoS ONE 7:*e*41722. 10.1371/journal.pone.0041722 22848578 PMC3404998

[B4] Andrizhiyevskaya E. G. Chojnicka A. Bautista J. A. Diner B. A. van Grondelle R. Dekker J. P. (2005). Origin of the F685 and F695 fluorescence in photosystem II. Photosynth. Res. 84, 173–180. 10.1007/s11120-005-0478-7 16049771

[B5] Bailey S. Thompson E. Nixon P. J. Horton P. Mullineaux C. W. Robinson C.. (2002). A critical role for the Var2 FtsH homologue of *Arabidopsis thaliana* in the photosystem II repair cycle *in vivo*. J. Biol. Chem. 277, 2006–2011. 10.1074/jbc.M105878200 11717304

[B6] Belgio E. Johnson M. P. Juric S. Ruban A. V. (2012). Higher plant photosystem II light-harvesting antenna, not the reaction center, determines the excited-state lifetime – both the maximum and the non-photochemically quenched. Biophys. J. 102, 2761–2771. 10.1016/j.bpj.2012.05.004 22735526 PMC3379028

[B7] Boehm M. Yu J. Krynicka V. Barker M. Tichy M. Komenda J. (2012). Subunit organisation of a *Synechocystis* hetero-oligomeric thylakoid FtsH complex involved in photosystem II repair. Plant Cell 24, 3669–3683. 10.1105/tpc.112.100891 22991268 PMC3480294

[B8] Burrows P. A. Sazanov L. A. Svab Z. Maliga P. Nixon P. J. (1998). Identification of a functional respiratory complex in chloroplasts through analysis of tobacco mutants containing disrupted plastid *ndh* genes. EMBO J. 17, 868–876. 10.1093/emboj/17.4.868 9463365 PMC1170436

[B9] Caffarri S. Kouril R. Kereïche S. Boekema E. J. Croce R. (2009). Functional architecture of higher plant photosystem II supercomplexes. EMBO J. 28, 3052–3063. 10.1038/emboj.2009.232 19696744 PMC2760109

[B10] Chiba S. Akiyama Y. Ito K. (2002). Membrane protein degradation by FtsH can be initiated from either end. J. Bacteriol. 184, 4775–4782. 10.1128/JB.184.17.4775-4782.2002 12169602 PMC135282

[B11] Chiba S. Akiyama Y. Mori H. Matsuo E.-I. Ito K. (2000). Length recognition at the N-terminal tail for the initiation of FtsH-mediated proteolysis. EMBO Rep. 1, 47–52. 10.1093/embo-reports/kvd005 11256624 PMC1083681

[B12] Ido K. Ifuku K. Yamamoto Y. Ishihara S. Murakami A. Takabe K. (2009). Knockdown of the PsbP protein does not prevent assembly of the dimeric PSII core complex but impairs accumulation of photosystem II supercomplexes in tobacco. Biochim. Biophys. Acta 1787, 873–881. 10.1016/j.bbabio.2009.03.004 19285950

[B13] Kato Y. Miura E. Ido K. Ifuku K. Sakamoto W. (2009). The variegated mutants lacking chloroplastic FtsHs are defective in D1 degradation and accumulate reactive oxygen species. Plant Physiol. 151, 1790–1801. 10.1104/pp.109.146589 19767385 PMC2785964

[B14] Kato Y. Sun X. Zhang L. Sakamoto W. (2012). Cooperative D1 degradation in the photosystem II repair mediated by chloroplastic proteases in Arabidopsis. Plant Physiol. 159, 1428–1439. 10.1104/pp.112.199042 22698923 PMC3425188

[B15] Komenda J. Barker M. Kuviková S. de Vrie R. Mullineaux C. W. Tichy M.. (2006). The FtsH protease slr0228 is important for quality control of photosystem II in the thylakoid membrane of *Synechocystis* sp. PCC 6803. J. Biol. Chem. 281, 1145–1151. 10.1074/jbc.M503852200 16286465

[B16] Komenda J. Knoppová J. Krynická V. Nixon P. J. Tichy M. (2010). Role of FtsH2 in the repair of photosystem II in mutants of the cyanobacterium *Synechocystis* PCC 6803 with impaired assembly or stability of the CaMn(4) cluster. Biochim. Biophys. Acta 1797, 566–575. 10.1016/j.bbabio.2010.02.006 20153291

[B17] Komenda J. Sobotka R. Nixon P. J. (2012). Assembling and maintaining the photosystem II complex in chloroplasts and cyanobacteria. Curr. Opin. Plant Biol. 15, 245–251. 10.1016/j.pbi.2012.01.017 22386092

[B18] Komenda J. Tichy M. Prásil O. Knoppova J. Kuvikova S. de Vries R.. (2007). The exposed N-terminal tail of the D1 subunit is required for rapid D1 degradation during photosystem II repair in *Synechocystis* sp PCC 6803. Plant Cell 19, 2839–2854. 10.1105/tpc.107.053868 17905897 PMC2048700

[B19] Kuroda H. Maliga P. (2001). Complementarity of the 16S rRNA penultimate stem with sequences downstream of the AUG destabilizes the plastid mRNAs. Nucl. Acids Res. 29, 970–975. 10.1093/nar/29.4.970 11160930 PMC29611

[B20] Lee S. Augustin S. Tatsuta T. Gerdes F. Langer T. Tsai F. T. F. (2011). Electron cryomicroscopy structure of a membrane–anchored mitochondrial AAA protease. J. Biol. Chem. 286, 4404–4411. 10.1074/jbc.M110.158741 21147776 PMC3039362

[B21] Michoux F. (2008). Developing New Strategies for the Production of Foreign Proteins in Higher Plant Chloroplasts. Ph.D. Thesis, Imperial College London.

[B22] Nixon P. J. Barker M. Boehm M. de Vrie R. Komenda J. (2005). FtsH-mediated repair of the photosystem II complex in response to light stress. J. Exp. Bot. 56, 357–363. 10.1093/jxb/eri021 15545296

[B23] Puthiyaveetil S. Kirchoff H. (2013). A phosphorylation map of the photosystem II supercomplex C2S2M2. Front. Plant Sci. 4:459. 10.3389/fpls.2013.00459 24298276 PMC3828554

[B24] Ruban A. V. Horton P. (1992). Mechanism of ΔpH-dependent dissipation of absorbed excitation energy by photosynthetic membranes. I. Spectroscopic analysis of isolated light harvesting complexes. Biochim. Biophys. Acta 1102, 30–38. 10.1016/0005-2728(92)90061-6

[B25] Silva P. Thompson E. Bailey S. Kruse O. Mullineaux C. W. Robinson C.. (2003). FtsH is involved in the early stages of repair of photosystem II in *Synechocystis* sp PCC 6803. Plant Cell 15, 2152–2164. 10.1105/tpc.012609 12953117 PMC181337

[B26] Suorsa M. Regel R. E. Paakkarinen V. Battchikova N. Herrmann R. G. Aro E.-M. (2004). Protein assembly of photosystem II and accumulation of subcomplexes in the absence of low molecular mass subunits PsbL and PsbJ. Eur. J. Biochem. 271, 96–107. 10.1046/j.1432-1033.2003.03906.x 14686923

[B27] Svab Z. Maliga P. (1993). High-frequency plastid transformation in tobacco by selection for a chimeric *aadA* gene. Proc. Natl. Acad. Sci. U.S.A. 90, 913–917. 10.1073/pnas.90.3.913 8381537 PMC45780

[B28] Torabi S. Umate P. Manavski N. Plöchinger M. Kleinknecht L. Bogireddi H.. (2014). PsbN is required for assembly of the photosystem II reaction center in *Nicotiana tabacum*. Plant Cell 26, 1183–1199. 10.1105/tpc.113.120444 24619613 PMC4001377

[B29] Vass I. (2012). Molecular mechanisms of photodamage in the photosystem II complex. Biochim. Biophys. Acta 1817, 209–217. 10.1016/j.bbabio.2011.04.014 21565163

[B30] Weis E. (1985). Chlorophyll fluorescence at 77 K in intact leaves: characterization of a technique to eliminate artefacts related to self absorption. Photosynth. Res. 6, 73–86. 10.1007/BF00029047 24442829

