# Supplementary figures and images for: Testing the Role of the N-Terminal Tail of D1 in the Maintenance of Photosystem II in Tobacco Chloroplasts
Source: Front Plant Sci. 2016 Jun 21;7:844. doi: 10.3389/fpls.2016.00844 (PMC4914591; doi:10.3389/fpls.2016.00844)

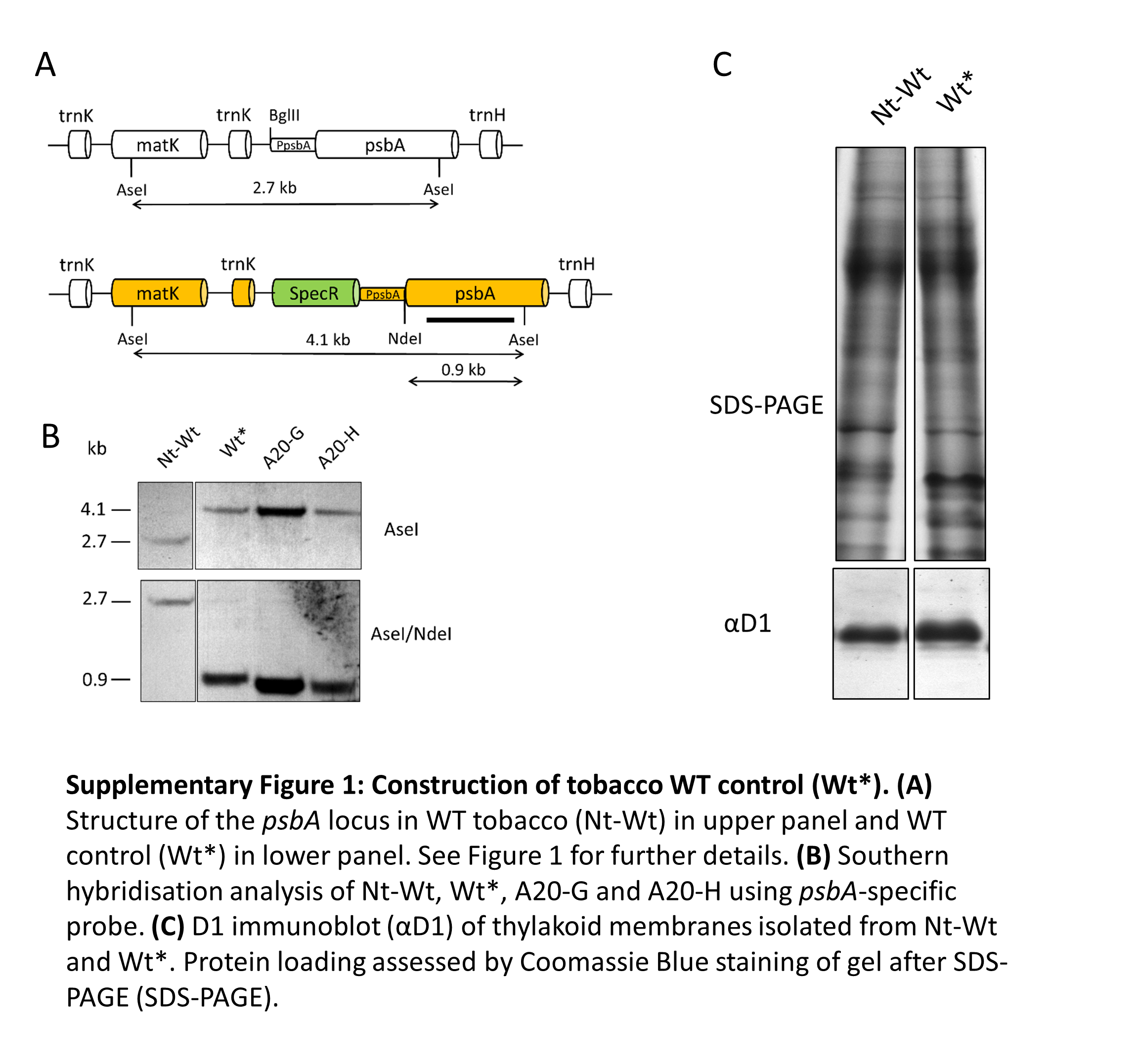

Supplement: Supplementary file 1 [file Image1.TIF]

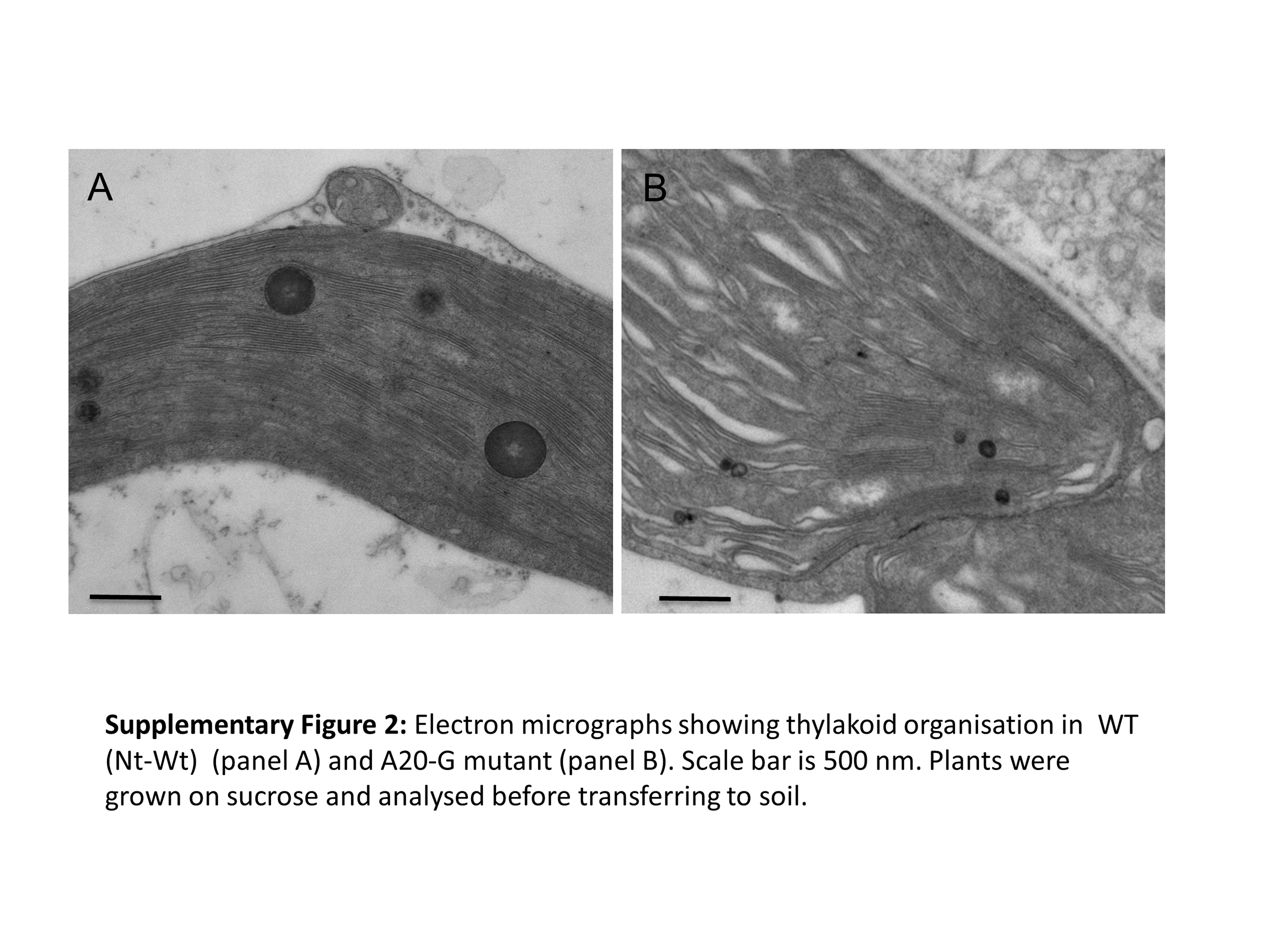

Supplement: Supplementary file 2 [file Image2.TIF]
